# Chronic meningoencephalomyelitis caused by *Nocardia nova* infection: a case report and literature review

**DOI:** 10.3389/fmed.2025.1680771

**Published:** 2025-12-04

**Authors:** Shao Li, Qianqian Tian, Xixi Yang, Mengyao Zhang, Menghan Zheng, Dawei Li, Zhihui Duan, Yan Li, Zhandong Qiu, Zheng Liu

**Affiliations:** 1 Department of Neurology, Xuanwu Hospital Capital Medical University, National Center for Neurological Disorders, Beijing, China; 2 Department of Neurology, Luoyang Central Hospital Affiliated to Zhengzhou University, Luoyang, China; 3 Department of Neurology, The Affiliated Hospital of Shandong Second Medical University, Weifang, China

**Keywords:** *Nocardia nova* infection, chronic meningoencephalomyelitis, targeted metagenomic next-generation sequencing, cerebrospinal fluid, central nervous system

## Abstract

A 25-year-old female zookeeper presented with 3-month history of sore throat and headache, 2-month intermittent fever, and 1-month dizziness. Neurological examination revealed bilateral nystagmus, left-sided sensory loss, ataxia, and subtle meningeal signs. Brain and cervical spinal cord MRI showed multiple enhancing lesions with central vein signs. Cerebrospinal fluid (CSF) analysis demonstrated elevated pressure (240 mmH2O) and leukocytosis (140 × 10^6^/L). Serum MOG-IgG was positive (1:32), while CSF metagenomic next-generation sequencing (mNGS) confirmed *Nocardia nova* infection. Initial treatment with trimethoprim-sulfamethoxazole (TMP-SMX), amikacin, and imipenem-cilastatin was followed by regimen adjustment to TMP-SMX plus minocycline at 6 weeks. One-month post-therapy, repeat CSF showed normalized pressure, reduced leukocytes, negative mNGS, and MRI evidence of lesion regression. Complete symptom resolution occurred 2 months after treatment initiation. This case exemplifies a rare presentation of *N. nova*-induced meningoencephalomyelitis with craniospinal involvement in an immunocompetent individual.

## Introduction

*Nocardia* is an aerobic, Gram-positive, and partially acid-fast pyogenic bacterium (1). Involvement of the central nervous system (CNS) represents a critical clinical event in disseminated infections, associated with high mortality and recurrence rates, especially in immunocompromised patients (2). *Nocardia*-induced meningoencephalomyelitis is exceedingly rare, with nonspecific symptoms and a high false-negative rates in conventional microbial cultures, resulting in limited literature on this condition. We report a case of isolated CNS nocardiosis presenting chronic meningoencephalomyelitis caused by *Nocardia nova* (*N. nova*) infection in an immunocompetent individual. The concurrent involvement of the brain and spinal cord, along with the presence of central venous signs, is extremely rare and has not been previously reported.

## Case presentation

We present a 25-year-old female patient admitted with chief complaints of “sore throat and headache for 3 months, intermittent fever for 2 months, and dizziness for 1 month.” Three months prior to admission, she developed upper respiratory infection symptoms including nasal congestion and sore throat, followed by headache predominantly involving bilateral temporal regions. Two months ago, fever occurred with the maximum body temperature reaching 38.5 °C, accompanied by aggravated headache. One month ago, she experienced dizziness, diplopia, numbness in the left limb and facial region, left hand tremor and weakness, while lower limb activities remained normal. Two days before admission, intermittent low-grade fever occurred with body temperature fluctuating between 37.5–38 °C. Neurological examination revealed bilateral horizontal nystagmus, decreased pinprick and tactile sensation on the left side of the body, unsteady bilateral finger-to-nose testing, inability to cooperate with the Romberg test, broad-based gait, and difficulty with tandem walking. Meningeal irritation signs were weakly positive. The patient works at a local zoo for 3 years, mainly responsible for taking caring of animals such as raccoons and elk.

**Figure 1:**
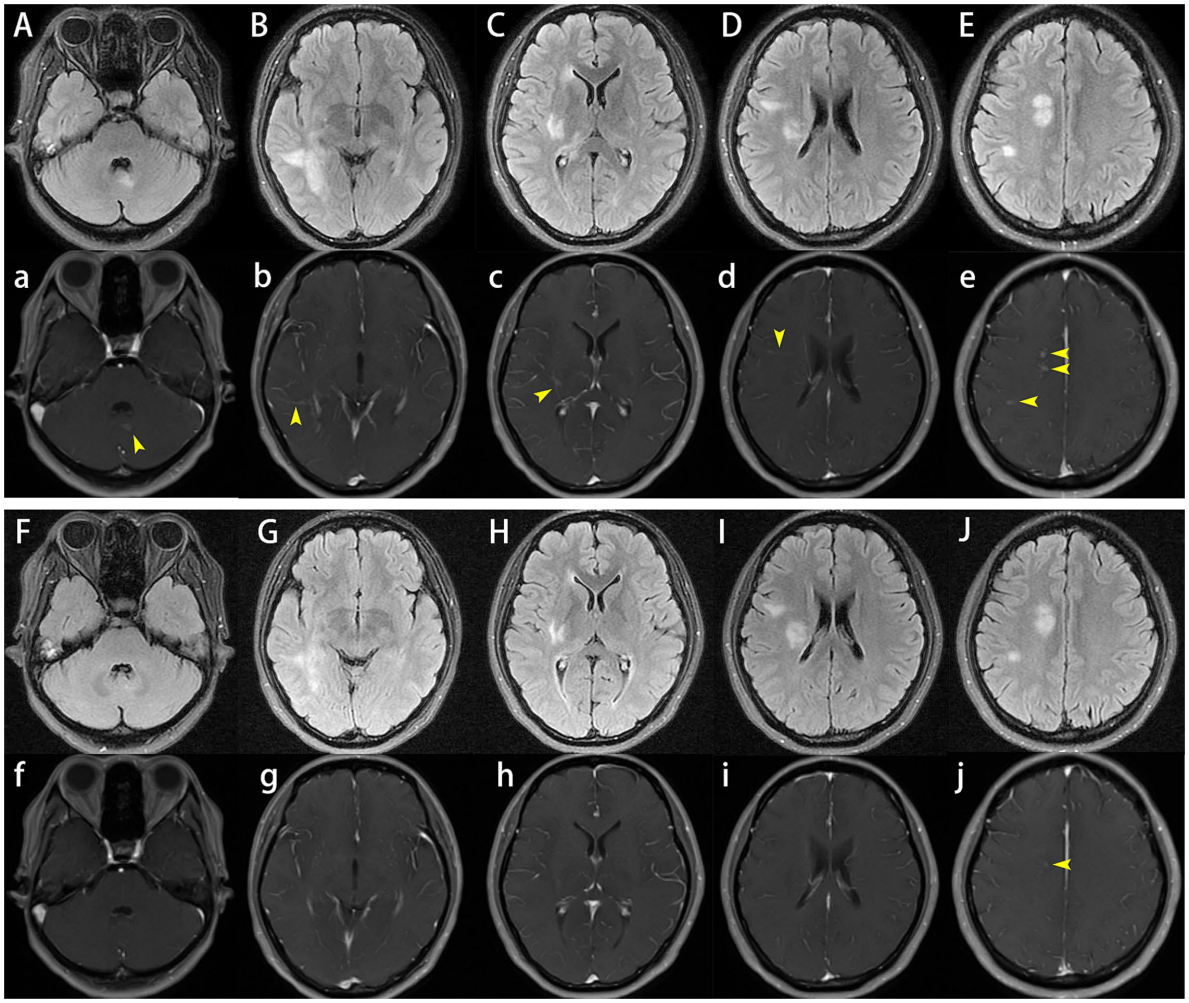
Cranial plain and enhanced magnetic resonance imaging (MRI). On admission, brain MRI demonstrated multiple abnormal signals in the intracranial region and cervical spinal cord, with nodular, patchy, or irregular enhancement **(A–E,a–e)**, the central vein sign was observed on T1-weighted enhanced sequences **(b–d)**. One month later, follow-up brain MRI showed that reduced lesion size and significant reduction or disappearance of enhancement **(F–J,f–j)**.

**Figure 2:**
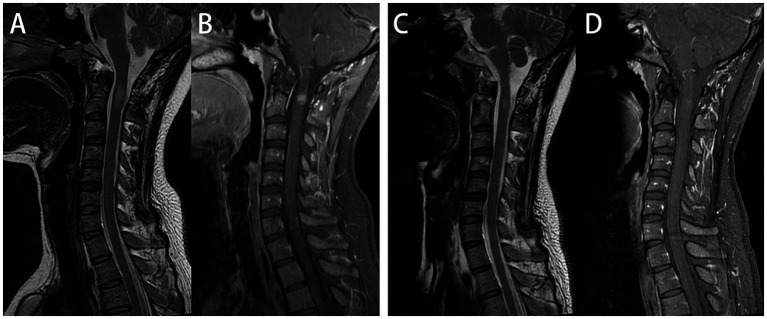
Cervical spinal cord plain and enhanced magnetic resonance imaging (MRI). On admission, cervical spine MRI demonstrated intramedullary abnormal signals at the C2 level, with nodular uniform enhancement **(A,B)**. One month later, follow-up cervical spine MRI showed that One month later, follow-up cervical spine MRI showed that a significant reduction in both the extent of the T2WI hyperintense and enhancing lesions **(C,D)**.

After admission, enhanced MRI of the brain and cervical spinal cord revealed multiple abnormal signals in the intracranial region and cervical spinal cord, with nodular, patchy, or irregular enhancement (Figures 1A–E,a–e, 2A,B). The central vein sign refers to the enhanced venous vascular shadow observed at the center of the lesion, surrounded by abnormal lesional tissue, which was detected on T1-weighted enhanced sequences (Figures 1b–d). Thoracic spinal cord MRI showed no significant abnormalities. Chest CT indicated bilateral minimal pleural effusion.

Given the gradual progression of neurological deficits, multiple CNS lesions, fever and work characteristics, differential diagnostic considerations included CNS infection, immune-mediated inflammatory demyelinating diseases, and central nervous system damage caused by systemic diseases. The initial investigation approach included routine blood tests and cerebrospinal fluid (CSF) analysis. The blood tests, erythrocyte sedimentation rate, C-reactive protein, antinuclear antibodies, antineutrophil cytoplasmic antibody, angiotensin-converting enzyme, etc. were all within normal ranges. T-SPOT. TB test and purified protein derivative (PPD) skin test were negative. CSF analysis showed a pressure of 240 mmH_2_O, white blood cell count of 140 × 10^6^/L (97% mononuclear, 3% polymorphonuclear), and normal protein and glucose levels. Serum and CSF antibodies tests for autoimmune encephalitis (anti-AMPAR1, AMPAR2, CASPR2, DPPX, GABABR, IgLON5, LGI1, NMDAR) and paraneoplastic syndromes (anti-Hu, Yo, Ri, Ma2/Ta, CV2/CRMP5, and amphiphysin, etc.), and CSF-specific oligoclonal bands were all negative. The test results for herpesviruses and parasitic antibodies in CSF, as well as DNA detection of herpesviruses types 1–8 and *Mycobacterium tuberculosis*, were all negative. In the detection of CNS demyelinating antibodies (anti-MOG, AQP4 and GFAP) in serum and CSF, her serum MOG-IgG was positive with a titer of 1:32. To better distinguish between infectious and inflammatory, metagenomic next-generation sequencing (mNGS) of CSF was performed, suggested *N. nova* infection (specific sequence count: 168, high confidence). The diagnosis was established as CNS infection caused by *N. nova*, with MOG-IgG likely representing post-infectious immune dysregulation or a bystander effect.

The patient was initiated on a therapeutic regimen consisting of oral trimethoprim-sulfamethoxazole (TMP-SMX) 1.44 g every 8 h, intravenous amikacin 0.8 g once daily, and intravenous imipenem-cilastatin sodium 0.5 g every 6 h. Notable gastrointestinal toxicities, including anorexia, nausea, and emesis, necessitated the addition of mosapride, pantoprazole, and vitamin B6, with subsequent resolution of these symptoms. Imipenem-cilastatin sodium was discontinued at 3 weeks, followed by amikacin discontinuation at 6 weeks. One month after therapy initiation, lumbar puncture revealed normal CSF pressure (170 mmH_2_O), mild leukocytosis at 15 × 10^6^/L (monocyte-predominant), and normal protein and glucose levels. Repeat CSF mNGS yielded negative results, whereas serum MOG-IgG remained persistently positive with stable titers. Serial cranial and cervical spinal MRI demonstrated reduction in lesion size and marked attenuation or resolution of enhancement (Figures 1F–J,f,g, 2C,D). At the 6-week treatment milestone, the regimen was transitioned to oral TMP-SMX in combination with minocycline 100 mg twice daily, with recommendations for continuation for a minimum of 12 months. Complete clinical remission was achieved 2 months following treatment initiation.

## Discussion

*Nocardia* is a group of aerobic, branching, Gram-positive, and partially acid-fast bacilli that are widely distributed in soil and vegetation, with relatively low pathogenicity (3). Although traditionally considered an opportunistic pathogen, up to one-third of cases occur in immunocompetent individuals, and male patients are significantly more numerous than female patients (2). *Nocardia* infections may present as either localized or disseminated forms, involving multiple systems including the skin and soft tissues, respiratory system, bones and joints, circulatory system, and CNS (4, 5). Nocardiosis with CNS involvement accounts for 3–26% of cases, with brain abscesses as the most common imaging manifestation, followed by leptomeningeal enhancement (2). The present case involves a young immunocompetent woman. Although febrile episodes were documented in small animals during her tenure at the zoo, and the institution had implemented protocols for isolation, diagnostic testing, and therapeutic intervention, no confirmed cases of nocardial infection in animals were reported within the facility. Consequently, establishing a definitive link between her infection and prolonged occupational animal exposure remains challenging. Notably, she exhibited no evidence of pulmonary abscess or cutaneous infection, with CNS involvement as the sole clinical manifestation.

Currently, more than 30 species of *Nocardia* are of clinical significance in humans, among which *N. nova* and *Nocardia farcinica* are the most common pathogens causing CNS infections. Most cases occur in patients with long-term use of corticosteroids or immunosuppressants, diabetes mellitus, human immunodeficiency virus (HIV) infection, malignancies, or organ transplantation (2, 6–8). The proportion of immunocompromised and transplant recipients was higher in disseminated disease compared to isolated CNS nocardiosis (2). The literature primarily described pulmonary infections caused by *N. nova*, followed by disseminated infections, which often involve the CNS in the form of brain abscesses or meningitis. Imaging findings may include leptomeningeal enhancement, single or multiple abscesses, and ventriculitis. Supratentorial abscesses are more common than infratentorial ones, while spinal cord involvement was exceedingly rare (2, 9–11), accounting for approximately 6.8% of patients with CNS involvement; the thoracolumbar spine was the most common site involved (2). The literature has previously reported a case of *Nocardia* infection involving the conus medullaris causing acute spinal cord compression (12). To our knowledge, there have been no reported cases of Nocardia infection simultaneously involving both the brain and spinal cord. In this case, the bilateral cerebral hemispheres and cervical spinal cord exhibited concurrent involvement, with imaging features of multiple lesions demonstrating homogeneous enhancement and central venous signs, rather than typical abscess characteristics. Post-therapeutic assessment revealed substantial regression in both lesion size and enhancement intensity. The relatively indolent clinical course in this patient is plausibly attributed to her intact immunocompetence and the implementation of early, aggressive therapeutic intervention.

Clinically, numerous diseases can cause intracerebral and spinal cord lesions, including infectious diseases, demyelinating diseases, and neoplastic diseases. In this case, the patient had a positive serum MOG-IgG, requiring differentiation from MOG-IgG-related disease (MOGAD). The clinical manifestations of MOGAD are diverse, with brain lesions typically appearing as a few “fluffy,” poorly defined, or large T2WI hyperintense signals. Longitudinal extensive transverse myelitis is relatively common (13). Our patient had a low serum MOG antibody titer, relatively well-defined lesions, and short-segment cervical spinal cord involvement with uniform nodular enhancement, and the symptoms significantly improved after anti-infective treatment alone, all of which are not supportive of a MOGAD diagnosis. We considered the possibility of a false-positive result or infection-triggered bystander effects.

Molecular biology techniques (such as metagenomic sequencing) can improve the efficiency of bacterial species identification, especially for cases with negative culture results or mixed infections. A study has shown that 43.7% of patients had bacterial species identified via polymerase chain reaction (PCR), which compensates for the limitations of traditional culture methods (2). Another study reported on nine patients in a single center, all of whom were diagnosed using mNGS (11). In recent years, an increasing number of patients have applied mNGS to the diagnosis and differential diagnosis of infectious diseases, playing an increasingly important role.

There are no standard recommendations for nocardiosis treatment. Individualized treatment regimens should be formulated based on the patient’s disease type, severity, comorbidities, and antimicrobial susceptibility testing. Most authoritative institutions recommend including trimethoprim-sulfamethoxazole (TMP-SMX) in the first-line treatment of nocardiosis (14, 15). Meena et al. (2) summarized and analyzed 129 papers and mentioned that TMP-SMX and amikacin are the most effective drugs for treating CNS nocardiosis. In clinical practice, patients with central CNS nocardiosis are generally recommended to receive an initial antimicrobial therapeutic regimen incorporating two or more agents—with trimethoprim-sulfamethoxazole (TMP-SMX) as a core component (11)—which typically consists of 3–6 weeks or longer of intravenous antimicrobial therapy followed by 1 year or more of oral antimicrobial treatment (16), though large-scale studies on the optimal antibiotic regimen and duration remain lacking. The patient was initially on an aggressive combination regimen consisting of TMP-SMX, amikacin and imipenem-cilastatin sodium, which elicited a substantial clinical response with marked symptom improvement. Subsequent management entails continuation of oral TMP-SMX and minocycline for a minimum of 1 year, coupled with scheduled cranial and spinal MRI to serially evaluate lesion dynamics.

Overall, nocardia-induced meningoencephalomyelitis represents an exceedingly rare clinical entity, particularly among immunocompetent individuals. The disease is characterized by a dismal prognosis, with a strikingly high case-fatality rate observed in the absence of timely diagnosis and intervention. Concomitantly, the therapeutic regimen for nocardiosis necessitates an extended treatment course, with combination antimicrobial therapy strongly advocated for central nervous system infections. Enhancing patient adherence to therapy emerges as a critical determinant in mitigating the risk of disease recurrence.

## Data Availability

The datasets presented in this study can be found in online repositories. The names of the repository/repositories and accession number(s) can be found in the article/supplementary material.
